# Interplay Between Depression and Inflammatory Bowel Disease: Shared Pathogenetic Mechanisms and Reciprocal Therapeutic Impacts—A Comprehensive Review

**DOI:** 10.3390/jcm14155522

**Published:** 2025-08-05

**Authors:** Amalia Di Petrillo, Agnese Favale, Sara Onali, Amit Kumar, Giuseppe Abbracciavento, Massimo Claudio Fantini

**Affiliations:** 1 Department of Medical Science and Public Health, University of Cagliari, 09042 Monserrato, Italysara.onali@unica.it (S.O.); massimoc.fantini@unica.it (M.C.F.); 2 Department of Electrical and Electronic Engineering, University of Cagliari, 09134 Cagliari, Italy; amit.kumar@unica.it; 3 Child Neuropsychiatric Unit, “A.Cao” Paediatric Hospital, 09121 Cagliari, Italy; g.abbracciavento@gmail.com

**Keywords:** depression, anxiety, inflammatory bowel disease, ulcerative colitis, Crohn disease, molecular mechanisms

## Abstract

Inflammatory bowel disease (IBD) is characterized by chronic inflammation of the gastrointestinal tract. Although the aetiology of IBD remains largely unknown, several studies suggest that an individual’s genetic susceptibility, external environmental factors, intestinal microbial flora, and immune responses are all factors involved in and functionally linked to the pathogenesis of IBD. Beyond the gastrointestinal manifestations, IBD patients frequently suffer from psychiatric comorbidities, particularly depression and anxiety. It remains unclear whether these disorders arise solely from reduced quality of life or whether they share overlapping biological mechanisms with IBD. This review aims to explore the bidirectional relationship between IBD and depressive disorders (DDs), with a focus on four key shared mechanisms: immune dysregulation, genetic susceptibility, alterations in gut microbiota composition, and dysfunction of the hypothalamic–pituitary–adrenal (HPA) axis. By examining recent literature, we highlight how these interconnected systems may contribute to both intestinal inflammation and mood disturbances. Furthermore, we discuss the reciprocal pharmacologic interactions between IBD and DDs: treatments for IBD, such as TNF-alpha and integrin inhibitors, have demonstrated effects on mood and anxiety symptoms, while certain antidepressants appear to exert independent anti-inflammatory properties, potentially reducing the risk or severity of IBD. Overall, this review underscores the need for a multidisciplinary approach to the care of IBD patients, integrating psychological and gastroenterological assessment. A better understanding of the shared pathophysiology may help refine therapeutic strategies and support the development of personalized, gut–brain-targeted interventions.

## 1. Introduction

Chronic inflammation of the gastrointestinal (GI) tract is the main feature of inflammatory bowel disease (IBD), which includes ulcerative colitis (UC) and Crohn’s disease (CD). The global prevalence of IBD is steadily increasing, with over 7 million individuals currently affected worldwide [1]. Many lines of evidence indicate a multifaceted interplay involving genetic predisposition, external environment, intestinal microbial flora, and immunological responses in the intricate pathogenesis of IBD [2].

Symptoms of IBD include diarrhoea, rectal bleeding, abdominal pain, exhaustion, and weight loss, with a characteristic relapsing–remitting course [3]. Notably, several studies have highlighted an elevated prevalence of mental comorbidity among IBD patients compared with the general population, particularly observing an increased incidence of anxiety and depression [4].

Diagnostic and Statistical Manual of Mental Disorders, Fifth Edition, Text Revision (DSM-5-TR) identifies depressive disorders (DDs) based on specific symptoms, namely, presence of sad, empty, or irritable mood, accompanied by related changes that significantly affect the individual’s capacity to function (e.g., somatic and cognitive changes in major depressive disorder and persistent depressive disorder [5]). To diagnose major depression, at least five of the following symptoms are required: depressed mood, loss of interest and pleasure in almost all activities, significant weight loss or gain, psychomotor agitation or retardation, chronic fatigue and loss of energy, feelings of worthlessness or excessive or inappropriate guilt, insomnia or hypersomnia, diminished ability to think or concentrate, and recurrent thoughts of death [5].

Similarly to that of IBD, the aetiopathogenesis of DDs is influenced by various factors that may act at different levels, such as psychological, biochemical, genetic, and social factors [6].

IBD patients are at an increased risk of suffering from mental disorders such as DDs as a result of the disease’s chronicity and the required changes in coping and self-management abilities over time. However, intriguingly, an increased incidence of depression and anxiety was reported in the years preceding the diagnosis of IBD, suggesting that psychiatric comorbidity is not only a psychological response to a chronic disease but could be due to the dysregulation of physiologic axes shared by both conditions [7,8].

A recent retrospective cohort study revealed that DDs increase the likelihood of developing IBD, and the use of antidepressants has been associated with a decreased risk of IBD [7]. Conversely, medications commonly employed for IBD have shown efficacy in reducing depressive symptoms [9].

Beyond this bidirectional association, identifying shared biological processes might be crucial to demonstrate this relationship. Studies exploring the mechanisms involved in the so-called gut–brain axis and their implication in the possible interconnection between chronic intestinal inflammation and susceptibility to DDs go in this direction [10]. In addition, both DDs and IBD share several physiopathological alterations that may explain their linkage, including immune system dysregulation, increased oxidative stress, the generation of proinflammatory metabolites, and genetic mutations [11].

Given the substantial global burden associated with both IBD and depressive disorders in terms of healthcare costs, reduced quality of life, and productivity loss, investigating the interplay between these conditions is of critical clinical and societal relevance.

The purpose of this review is to explore the intricate connection between IBD and DDs, highlighting the reciprocal influence between these two conditions. Specifically, we examined four major shared pathogenetic mechanisms: (1) genetic susceptibility, (2) gut microbiota dysbiosis, (3) immune system dysregulation, and (4) disturbances of the hypothalamic–pituitary–adrenal (HPA) axis. We also reviewed available data on the bidirectional pharmacological effects, exploring how treatments for DDs may influence the risk or course of IBD and vice versa. Finally, this review aims to provide a conceptual framework to guide future research toward the identification of common mechanisms that could support the development of integrated and multidisciplinary therapeutic strategies for both DDs and IBD.

## 2. Psychiatric Comorbidity Prevalence and Incidence in IBD

Several studies have demonstrated a higher prevalence of psychiatric illnesses among IBD patients compared with the general population [12,13]. In a retrospective US cohort including 393 IBD patients without previous mental diagnosis, the incidence of DDs was 20.1% [4]. When considering data from a large Canadian health survey, it was observed that the prevalence of DDs in individuals with IBD ranged between 14.7% and 16.3% [14]. European surveys have indicated lower DDs rates, with a prevalence of 10.2% in Germany [15] and 10% in France among IBD patients [4,16]. The anxiety levels are even greater, ranging from 29–35% during remission to 80% during exacerbation [16]. In the Manitoba IBD Cohort research, a comparison of lifespan prevalence indicated that IBD patients had greater rates of major depression than the general population (27.2% vs. 12.3%, OR 2.20, 95% CI 1.64–2.95), but not of panic disorders (8.0% vs. 4.7%, OR 1.59, 95% CI 0.96–2.63) [17].

A recent systematic review and meta-analysis, in which the authors performed subgroup analyses by gender, disease location, and disease activity, country, and method used to define anxiety and depression, revealed that among IBD patients, there was a high prevalence of anxiety and depression symptoms, with approximately one-third of patients experiencing anxiety symptoms and one-quarter experiencing depression symptoms [18]. However, it is important to consider that the prevalence of depression in IBD may be underreported, as symptoms such as fatigue, sleep disturbance, or somatic complaints can overlap with those of active intestinal disease or anxiety, potentially leading to misdiagnosis or diagnostic delay. The diagnostic complexity of DDs may contribute to the variability observed across studies and highlight the need for standardized screening protocols in clinical practice.

In a case-control study conducted in the south of England, a similar tendency was noted; this study suggested that the risk of depression and anxiety is maximal during the initial year after IBD diagnosis [8]. However, in the same study, depression and anxiety were more frequent in UC (but not CD) patients when the diagnosis of IBD followed that of DD by 5 or more years. Using data from an administrative health data record, Berstain et al. also demonstrated after adjusting for age, area of residence, sex, and year, there was higher incidence of depression (incidence rate ratio [IRR], 1.58; 95% confidence interval [CI], 1.41–1.76), anxiety disorder (IRR, 1.39; 95% CI, 1.26–1.53), and bipolar disorder (IRR, 1.82; 95% CI, 1.44–2.30) among IBD patients compared with controls [12].

In a prospective cohort study conducted in the UK, patients with IBD exhibited a 23% higher risk of developing overall psychiatric disorders compared with non-IBD controls [adjusted HR (aHR) 1.23, 95% CI: 1.13–1.33, *p* < 0.001]. The increased risk was consistent for depression (aHR 1.36, 95% CI: 1.22–1.52, *p* < 0.001), anxiety (aHR 1.15, 95% CI: 1.01–1.30, *p* = 0.031), and post-traumatic stress disorder (PTSD) (aHR 1.87, 95% CI: 1.00–3.51, *p* = 0.047). Notably, patients with CD (aHR 1.47, 95% CI: 1.23–1.76, *p* < 0.001), but not UC (aHR 1.01, 95% CI: 0.84–1.21, *p* = 0.901), showed a significantly higher risk of being affected by psychiatric disorders compared with non-IBD controls [19]. Moreover, a recent systematic review and meta-analysis by Gong et al. found a significant association between symptoms of depression and an increased risk of disease flare in IBD patients (OR 1.69; 95% CI 1.34–2.13) [20].

## 3. Shared Molecular Mechanisms

**Figure 1:**
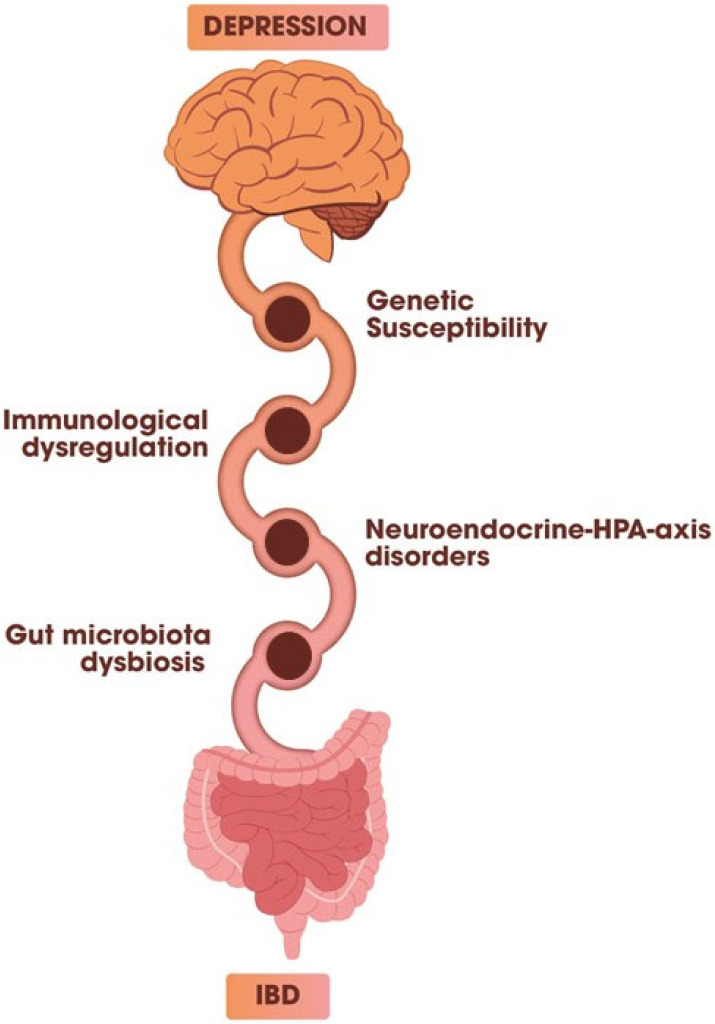
Schematic representation of the main pathogenetic mechanisms shared between inflammatory bowel disease (IBD) and depressive disorders (DDs). The bidirectional connection between the brain and the gut is mediated by several overlapping biological pathways, including (1) genetic susceptibility, (2) immune system dysregulation, (3) alterations in the hypothalamic–pituitary–adrenal (HPA) axis, and (4) gut microbiota dysbiosis. These mechanisms contribute to the comorbidity of IBD and depression by influencing both intestinal inflammation and central nervous system function.

IBD and DDs share multiple pathophysiological alterations (Figure 1), including elevation of C-reactive protein (CRP) and proinflammatory cytokines, increased intestinal permeability, dysbiosis, and oxidative stress [21]. Recent studies have postulated bidirectional communication between the brain and gut microbiota, making the microbiota–gut–brain axis the major link between psychological distress and IBD [10].

### 3.1. Genetic Susceptibility

IBD is a complex, multifaceted, polygenic disease that primarily affects the activity of the gut-associated immune system. Genome-wide association studies (GWASs) have identified more than 200 susceptibility loci associated with key signalling pathways crucial for intestinal homeostasis, such as antimicrobial peptide secretion, barrier function, control of innate immunity [22,23], oxidative stress [24], intracellular signal transduction [25], and autophagy [26].

New genetic evidence supports the concept that immunological pathways contribute to the aetiology of both DDs and IBD.

The first identified IBD susceptibility gene was the nucleotide-binding oligomerization domain containing 2 (NOD2). Three NOD2 allelic variants are strongly associated with Crohn’s (ileal) disease [27]: R702W, G908R, and 3020insC. NOD2 is a key factor in the regulation of the microbiota in the small intestine via antibacterial secretions [28]. Additionally, allele variants of other genes involved in the regulation of the innate and adaptive immune systems, such as HLA, IL23R, STAT3, JAK2, were shown to increase the risk of developing IBD [29].

In the context of DDs, an analysis of 44 single-nucleotide polymorphisms (SNPs) from GWAS revealed the enrichment of 19 pathways, including those associated with immune response and proinflammatory cytokine production [30]. In a Mendelian randomization study involving candidate genes, a functional SNP in the IL-6 receptor promoter was identified, providing estimates of CRP, IL-6, and the severity of depression symptoms in a population cohort study [31]. Furthermore, a combined GWAS and investigation into human brain proteomics found that genetic alterations in P2RX7, which encodes for the purinergic receptor P2X7, played a role in the development of DDs [32]. This genetic modulation of P2X7 has broader implications, as it inhibits the activation of the NOD-, LRR-, and pyrin domain-containing 3 (NLRP3) inflammasome complex, a key modulator of innate immune activation and involved in IBD pathogenesis [33].

A loss-of-function SNP variant in the PTPN2 gene, associated with T cell signaling and immunological functions, has been linked to both IBD and DDs. In T cells, PTPN2 attenuates T cell antigen receptor (TCR) signaling by dephosphorylating the TCR-associated kinases. This gene plays an important role in several immunological functions, including T cell differentiation, intestinal epithelial homeostasis, and cytokine signaling modulation [34]. In PTPN2 knockout murine model, lower insulin secretion and significantly decreased release of norepinephrine, dopamine, and 5-hydroxytryptamine function in the brain were observed, which led to changes in anxiety-like behavior [34,35,36,37,38].

Lasconi et al. [39] highlighted the possible genetic connections between IBD and DDs, with a focus on the role of the hypothalamus and the hypothalamic–pituitary–adrenal (HPA) axis, by analysing publicly available GWAS data on depression and IBD traits. They identified significant genetic correlations between IBD and depression, suggesting shared genetic factors between the two conditions and indicating a potential genetic link between IBD and mood disorders. They observed the enrichment of IBD-associated genetic variants in open chromatin regions of hypothalamic-like neurons and colonoids, suggesting the potential involvement of the hypothalamus in the genetic predisposition to IBD. Among IBD susceptibility genes, cAMP-responsive element modulator (CREM), ciliary neurotrophic factor (CNTF), and Ras homolog family member A (RHOA) were identified in hypothalamic cells. These genes are known to play roles in stress response pathways. For instance, CREM encodes a protein involved in regulating stress response pathways, while CNTF is a neurotrophic factor released in response to acute stress and necessary for the synthesis of cortical norepinephrine, which modulates stress responses and the HPA axis. RHOA, meanwhile, regulates social stress responses and depressive-like behaviour by mediating dendritic remodelling of neurons responsible for motivation and reward.

A recent study identified four key genes associated with both IBD and major depressive disorder (MDD) using a combined analysis of gene expression data and proteomic evaluation. Hepatocyte growth factor (HGF), secreted protein acidic and cysteine-rich (SPARC), a disintegrin and metalloproteinase 12 (ADAM12), and matrix metallopeptidase 8 (MMP8) were identified as biomarkers with significant roles in inflammatory processes and immune dysregulation, contributing to both IBD and MDDs. The identification of these genes provides new insights into the molecular pathways linking IBD with psychiatric disorders, further supporting the concept of shared immunological mechanisms underlying these diseases [40].

In line with these findings, Tripathi et al. used GWAS meta-analysis and GTEx gene expression data to identify a shared genetic architecture between IBD and several neuropsychiatric disorders, including depression. Notably, many IBD-associated genes, such as IL23R, NOD2, and ATG16L1, were expressed in specific regions of the brain, suggesting their systemic involvement and the relevance of neuroimmune communication in disease pathogenesis [41].

Additionally, Pinakhina et al. identified the MAGI2 (S-SCAM) gene as a potential susceptibility gene for depression. This gene exhibits both gut and brain expression patterns, reinforcing its functional role in the gut–brain axis and the potential convergence of pathways implicated in both IBD and DDs [42].

In summary, these findings provide valuable insights into the genetic contribution to the pathogenetic mechanisms operating in IBD and DDs, shedding light on potential pathways and fostering further research for new therapeutic interventions [43].

### 3.2. Gut Microbiota Dysbiosis

There are around 100 trillion fungi, bacteria, protozoa, and viruses in the human digestive tract. More than 99 percent of these bacteria belong to the phyla *Firmicutes*, *Proteus*, *Bacteroides*, and *Actinomycetes*, with *Bacteroides* and *Firmicutes* dominating the host’s normal gut flora. Microbiota serves a specific function, such as food absorption, gastrointestinal digestion improvement, and intestinal integrity maintenance [44].

There is a symbiotic relationship between gut microbiota and the host, which provides the host with numerous health benefits [45,46], and alteration of this relationship plays a pivotal role in the pathophysiology of IBD. Active IBD patients have been shown to have fewer butyrate-producing gut bacteria than healthy controls. Specifically, the levels of *Bifidobacterium longum*, *Eubacterium rectale*, *Faecalibacterium prausnitzii*, *Roseburia intestinalis*, and other beneficial bacteria were significantly reduced in both CD and UC, while the relative abundance and growth rate of harmful bacteria such as *Bacteroides fragilis* were enhanced [47]. *Christensenellaceae*, *Coriobacteriaceae*, and especially *Clostridium leptum* are less prevalent in CD patients, whereas *Actinomyces* spp., *Veillonella* spp., and *Escherichia coli* are more prevalent. *Eubacterium rectum* and *Escherichia coli* are increased in UC patients, whereas *Akkermansia muciniphila* levels are significantly reduced [48]. Although *Christensenellaceae and Clostridium leptum* are generally regarded as beneficial commensals associated with gut homeostasis, recent studies suggest that they may exhibit dual behavior, potentially contributing to inflammation under certain IBD phenotypes or disease states [49]. This highlights the complexity of microbiota–host interactions and the need for careful interpretation of microbial shifts in the context of clinical care.

The intestinal microbiota is also known to regulate psychological functions, such as anxiety and depression. Several studies and meta-analyses indicated that the genera *Corprococcus* and *Faecalibacterium* were reduced in depressed patients compared with nondepressed controls and that probiotic intervention studies relieved depressive symptoms [50,51]. According to a meta-analysis, patients with psychiatric disorders, including DDs, have a transdiagnostic pattern characterised by the depletion of certain anti-inflammatory butyrate-producing bacteria, while proinflammatory bacteria are increased [52,53].

A recent study conducted on a cohort of 507 IBD patients and 75 controls identified 106 differentially abundant taxa (DATs) in IBD patients compared with controls, and 21 DATs that distinguished IBD patients with coexisting mental disorders from those without. Among these, reductions in beneficial bacteria such as *Faecalibacterium prausnitzii*, *Roseburia intestinalis*, and *Bifidobacterium longum* were confirmed, reinforcing previous findings. Conversely, higher levels of *Escherichia coli*, *Veillonella* spp., and *Actinomyces* spp., known to be proinflammatory, were more prevalent in IBD patients with mental health comorbidities. The study also demonstrated that IBD patients with mental disorders had significantly reduced microbial diversity. These results support the notion that gut microbiota dysbiosis may play a crucial role in the link between IBD and mental health disorders, suggesting a potential therapeutic target via modulation of the microbiota–gut–brain axis [54].

Emerging evidence suggests that microbial metabolites, such as short-chain fatty acids (SCFAs) and bile acids, play a significant role in modulating intestinal immunity, barrier integrity, and even mood, through their impact on the gut–brain axis. However, most existing studies focus on microbial composition rather than function. This functional dimension of the microbiota warrants further investigation.

Recent advances have also explored strategies based on engineered probiotics to restore the intestinal microenvironment. One innovative platform (ZI@EcN), based on *Escherichia coli* Nissle 1917 functionalized with zinc and indole-3-carbinol, was shown to repair the epithelial barrier, reduce inflammation, and promote recolonization by beneficial bacteria in animal models of colitis [55].

With a different approach, mucoadhesive “probiotic backpacks” carrying ROS-scavenging nanoparticles (HPN-NE-EcN) demonstrated enhanced probiotic delivery, reduction of oxidative stress, and microbiome recovery in inflamed colonic tissue [56]. These strategies highlight the potential of next-generation probiotics not only in IBD therapy but also for modulating mood via microbiota–immune–brain pathways.

In addition, reconstitution of a eubiotic equilibrium using microorganisms or faecal microbiota transplant (FMT) may be an effective treatment in patients suffering from DDs. Studies exploring the effectiveness of FMT in the treatment of depression are reviewed in [57]. Multiple studies and case reports suggest that FMT might alleviates depression symptoms in depressed patients [58,59,60,61,62,63,64,65]. In a recent study, FMT from individuals with IBD and DDs induced sadness and gut inflammation in mice without the presence of stress [66,67] by inhibiting IL-1β and IL-6 expression while the administration of probiotics reduced inflammation [68].

### 3.3. Immunological Dysregulation

The patrolling activity of the immune system in the gut against pathogens and pathobionts involves various cellular types, including dendritic cells (DC), macrophages, natural killer cells (NK), natural killer T cells, innate lymphoid cells, and intraepithelial lymphocytes. Also functionally linked to the immune-patrolling activity is the physical/chemical barrier composed by IEL, Paneth cells, and microfold cells (M cells). In IBD, an aberrant immune response against microbiota-derived antigens normally contained in the gut lumen is initiated by DCs, which capture and recognize antigens by projecting their dendrites into the intestinal lumen between intestinal epithelial cells. Alternatively, antigens become available to DCs through M cells located on the surface of Payer’s patches and lymphoid follicles. Subsequently, DCs migrate to mesenteric lymph nodes and present antigens to naive T cells (Th0) [69].

Antigen presentation in the presence of the cytokine interleukin (IL)-12 or a combination of IL-1beta, IL-6, and transforming growth factor-beta (TGF-beta) leads to the polarization of Th0 cells into a proinflammatory Th1 or Th17 profile, respectively. These T cell phenotypes are increased at the expense of a relative reduction of regulatory T cells (Tregs), T cells with suppressive and anti-inflammatory activity. Th1 and Th17 cells secrete cytokines such as tumour necrosis factor (TNF)-alpha, interferon (IFN)-gamma, IL17A, IL-17F, and IL-21, contributing to tissue damage and an excessive inflammatory response, especially in CD.

Furthermore, cytokines such as IL-4, IL-33, IL-25, and thymic stromal lymphopoietin directly or indirectly contribute to the differentiation of Th0 cells into the Th2 profile in UC, which may also be under the control of Tregs. Dysregulation of the immune response in the intestine is a crucial factor in the pathogenesis of IBD [70].

Depressive patients have significantly higher levels of the proinflammatory cytokines IL-1, IL-6, TNF, and CRP compared with healthy individuals [71]. Numerous preclinical and clinical data show that Th17 and Treg cells contribute considerably to DDs [72,73,74,75,76,77]. Indeed, “the macrophage theory of depression” [78] had already been postulated three decades ago.

As in the intestinal context, where the balance between Th17 and Treg cells is crucial, similarly, brain-associated Th17 and Treg cells maintain immunological homeostasis for neuroinflammation associated with age, microglial activation, astrocytic activation, and brain development during pregnancy [79,80,81,82].

A tight connection between intestinal Th17/Treg balance and DDs has been suggested in preclinical studies. In a murine model, expansion of Tregs in the gut mucosa induced by the administration of probiotics and prebiotics through the modulation of ILC3 cells, induced behavioral resilience to chronic and recurrent stress, while impairment of Th17/Treg balance was associated with hippocampal chemotactic-chemokine and prefrontal cortex IL1b production in the context of stress-induced behavioral deficits [83]. In another experimental model, quorum-sensing AI-2 produced by segmented filamentous bacteria (SFB) promoted depressive-like behavior, and this phenomenon was dependent on the expansion of Th17 cells in the gut and hippocampus in response to endogenous serum amyloid proteins (SAA) 1 and 2 induced by AI-2-producing SFB [84].

Many studies exploring the relationship between gut microbiota and Treg cells have focused on SCFAs.

SCFAs can induce Tregs indirectly by butyrate-induced DCs and macrophages [85]. However, a direct effect of SCFAs on Treg proliferation and function has been demonstrated through GPR43 and histone deacetylase inhibition [86]. Finally, propionate and butyrate induce Treg differentiation by upregulating FoxP3 in a histone-acetylation-dependent manner [87,88]. In GPR43-induced human Tregs, butyrate increases IL-10 production, thus increasing the Tregs’ suppressive capacity [89]. It is also relevant to understand the complex balance between pro- and anti-inflammatory mechanisms operating in the gut mucosa. Indeed, depending on their concentration and the cytokine “atmosphere” generated, SCFAs can either promote the expansion of Tregs and enhance IL-10 expression or promote the differentiation of Th0 cells into proinflammatory Th1 or Th17 cells [90,91].

By generating polysaccharide A (PSA), *Bacteroides fragilis* induced Tregs and promoted expression of IL-10. At the same time, PSA reduces the induction of Th17 in a TLR2-dependent manner, thus unbalancing the Th17/Treg cell ratio in favour of Treg cells [92]. Clostridium IV and XIV bacteria stimulate the expression of Foxp3 and the formation of Treg cells through butyrate production and enhancement of histone H3 deacetylase activity [92].

Increased levels of peripheral proinflammatory cytokines, such as TNF-alpha and IL-6, have been observed in patients affected by DDs [93]. The functional link between proinflammatory cytokine expression and DDs is further supported by the finding that anti-TNFs ameliorate depression-like behaviours in psoriatic patients independently of skin lesion clearance or the regression of joint inflammation [94]. Accordingly, in a randomized double-blind control study, a single dose of ketamine infusion caused a significant decrease in serum TNF-alpha levels 40 min after infusion in patients with treatment-resistant depression, and this was associated with a reduction in depression symptoms as assessed by depressive rating scores [95].

Finally, O’Donovan et al. found that patients suffering from MDD with high suicidal ideation had significantly higher serum levels of IL-6 and CRP than MDD patients with lower suicidal ideation and control subjects [96].

The development of biological therapy involving monoclonal antibodies that selectively target dysregulated inflammatory mediators, and their efficacy in treating both IBD and DDs, indicates that immunological dysregulation is a shared pathogenetic mechanism underlying both disorders [97,98]. Table 1 provides a comprehensive overview of interleukin levels, both upregulated and downregulated, in the serum and/or mucosal intestinal tissues of individuals with IBD and DDs.

### 3.4. Hypothalamic–Pituitary–Adrenal (HPA) Disorders

The hypothalamus is a neuronal component of the limbic system that regulates the secretory activity of the pituitary gland, thus bridging the nervous and endocrine systems. In response to stress, the HPA axis is activated, causing the hypothalamus to produce corticotropin-releasing hormone (CRH) and the pituitary gland to produce adrenocorticotropic hormone (ACTH). In turn, ACTH stimulates the adrenal cortex to secrete cortisol, causing the peripheral production and release of anti-inflammatory cytokines. Moreover, the integration of stress signals in the hypothalamus results in the secretion of sympathetic neurotransmitters in the gut, as evidenced by the modification of intestinal motility, permeability, and secretory activity [21,124,125]. The stimulation of the HPA system following stress exposure may be one of the major mechanisms behind the development of DDs. Typically, depressed individuals demonstrate either elevated cortisol levels or decreased corticosteroid receptor activation.

While acute stress typically triggers an adaptive and transient HPA response, chronic stress leads to HPA axis dysregulation characterized by sustained hypercortisolemia, impaired glucocorticoid receptor sensitivity, and reduced negative feedback [126]. These alterations have been associated with hippocampal atrophy, emotional dysregulation, and increased susceptibility to depressive disorders [77]. Notably, up to fifty percent of depressed individuals exhibit excessive activation of the HPA axis [127].

In patients with IBD, chronic psychological stress has been shown to exacerbate intestinal inflammation, potentially through cortisol-mediated effects on gut barrier integrity, microbiota composition, and mucosal immune activation. Stress-induced intestinal permeability and microbial translocation may trigger sustained immune activation, perpetuating a vicious cycle between inflammation and mood disorders [128,129].

Furthermore, despite elevated systemic cortisol, colonic cortisol levels may be insufficient in IBD patients under chronic stress, contributing to local immune dysregulation and persistent inflammation. Clinically, high perceived stress levels have been associated with CD flares and may even precede disease onset, suggesting that stress acts not only as a consequence but as a contributing factor to IBD pathogenesis [126].

Evidence supporting the gut–brain connection was obtained in germ-free (GF) mice, where the gut microbiota was shown to regulate the HPA axis set point and behavioural stress responsiveness [130]. In contrast, sustained stress leads to HPA-axis dysfunction and colonic cortisol insufficiency, which may cause persistent colonic inflammation in susceptible hosts [131].

Finally, recent genomic studies have revealed a significant enrichment of IBD-associated single-nucleotide polymorphisms (SNPs) in open-chromatin regions of hypothalamic promoter-interacting neurons, highlighting the potential relevance of this brain region in the neuroimmune regulation of IBD [39].

These findings support the role of the hypothalamus in the pathophysiology of IBD.

## 4. Bidirectional Impact of Medications in Inflammatory Bowel Disease and Depressive Disorders

DDs and IBD share multiple pathogenetic pathways, including immunological disorders, dysbiosis, and HPA axis abnormalities. Numerous studies have shown that targeting these mechanisms improves both IBD and depressive symptoms.

### 4.1. Impact of Medications Used in IBD on DDs

In IBD, many advanced therapies are based on the use of monoclonal antibodies that selectively block dysregulated cytokines released by inflammatory cells. Few studies have examined the influence of IBD therapies, mainly TNF inhibitors, on depression and anxiety. Patients with active IBD treated with anti-TNFs demonstrated a reduction in depressive symptoms and anxiety, as well as an improvement in disease activity [132]. The CHARM trial investigated the efficacy of adalimumab (ADA) in CD. In this trial, the validated Zung self-rating depression scale was used to evaluate the presence of depressive symptoms among the studied population. At the end of the induction phase, the mean values of the Zung depression scale decreased significantly in ADA-treated patients, corresponding to a shift from mild depression to a normal-range score, while the same values worsened in the placebo group [133]. Interestingly, in a meta-analysis of 152 psychiatric patients affected by treatment-resistant depression, anti-TNFs were shown to have no effect in reducing depressive symptoms [134]. In a randomized, placebo-controlled, double-blind clinical trial conducted in patients affected by treatment-resistant depression, Raison et al. confirmed the inefficacy of anti-TNFs in reducing depression symptoms [135]. However, a subgroup analysis revealed that treatment response (defined as ≥50% reduction in the 17-item Hamilton Depression Rating Scale at any point during treatment) correlated with baseline elevated C-reactive protein (CRP) [62% (8/13) in the infliximab (IFX) group versus 33% (3/9) in placebo-treated patients (*p* = 0.19)]. This could in part explain why anti-TNFs have shown no efficacy in decreasing depressive symptomatology in the overall population of patients but improved depression symptoms in patients with evidence of high inflammatory burden.

In addition, one study found that the antialpha4/-beta7 integrin vedolizumab (VEDO) improved depression and anxiety symptoms in IBD patients to the same extent as anti-TNFs [9]. Accordingly, Stevens et al. demonstrated that both VEDO and anti-TNFs caused improvements in depression and anxiety as well as sleep quality within 6 weeks from therapy initiation and that such improvements were sustained up to one year [9].

On the other hand, data from two randomized clinical trials (RCTs) on CD patients treated with the anti p40IL12/IL23 antibody ustekinumab (USTE) and VEDO found depression as the second most common and the most common adverse psychiatric event (APE), respectively [136,137]. Although this seems confounding and in contrast with the previously mentioned data, biologic therapies have been shown to ameliorate depressive symptoms in patients who already had symptoms at baseline, possibly by reducing the inflammatory burden and demonstrating their known beneficial role in inflammatory-driven depression. On the contrary, depression is considered a therapy-related APE in those patients who had no psychiatric diagnosis at baseline, revealing a possible different mechanism through which these drugs may act starting from a different baseline psychiatric status.

Alternatively, depressive symptoms might initially be underestimated during active phases of IBD, as they are often attributed to the underlying intestinal inflammation. However, once clinical remission is achieved following therapy, the persistence of these symptoms, despite the resolution of intestinal disease, may prompt physicians to consider the presence of a previously unrecognized, masked depression. In such cases, the temporal association between treatment initiation and the emergence of depressive symptoms might raise the hypothesis that the treatment itself could play a contributory role. Although this does not imply a direct causal relationship, it remains a possibility that warrants further investigation (Table 2).

### 4.2. Impact of Depression Medications on IBD

The association between major depression and the activation of the inflammatory response is well documented. Similarly as in IBD patients, acute-phase proteins such as CRP are increased in patients affected by depression, while negative acute-phase proteins such as albumin are decreased [140]. Indeed, treatment with antidepressants leads to a drop in CRP concentrations independently of the resolution of depressive symptoms [140]. This finding led to the development of several studies aiming to investigate antidepressants’ effects on IBD patients (Table 3). On the other hand, being affected by major depressive disorder is a negative predictor of clinical remission after IFX treatment [141]. A retrospective study by Goodhand et al. [138] assessed the course of IBD one year before and one year after the initiation of antidepressant medications prescribed for mood disorders. The authors found that patients treated with antidepressants had fewer IBD relapses and less frequent need for steroids in the year before therapy initiation. The same results were found in another Danish nationwide study, where patients currently under antidepressive therapy had significantly lower relapse rates compared with those untreated, and this was more pronounced in CD than in UC patients [142]. Moreover, IBD patients taking antidepressants needed fewer step-up medications, such as steroids or anti-TNFs, and had fewer hospitalizations than nonusers [142]. Interestingly, in a retrospective cohort study, tricyclic antidepressants (TCA) were beneficial for IBD patients with residual abdominal symptoms despite quiescent or mild inflammatory activity [143]. Accordingly, duloxetine, in a placebo-controlled randomized clinical trial, was effective in reducing the severity of some physical symptoms in IBD patients, such as abdominal pain [144]. However, in an Australian online survey for IBD patients on antidepressant medications, the majority of responders observed no change in IBD disease activity while on treatment [145].

In addition, a recent study on IBD patients and matched non-IBD controls found that antidepressant use was independently associated with higher frequency of visits, corticosteroid use, and hospitalizations, while neither IBD-associated complications nor surgery were increased in antidepressant users [146].

A recent systematic review and meta-analysis further confirmed that antidepressants improve depressive symptoms and quality of life in IBD patients. Notably, serotonin and noradrenaline reuptake inhibitors (SNRIs) demonstrated particular efficacy in improving depression, anxiety, and overall well-being. These findings emphasize the potential role of SNRIs, such as duloxetine and venlafaxine, in improving psychiatric comorbidities in IBD patients [147].

Finally, animal models of IBD have shown that antidepressant medications can lower intestinal inflammation by regulating neurohumoral pathways [148,149,150,151].

Despite the accumulating evidence on the possible anti-inflammatory effect of antidepressant medications in IBD, a Cochrane review stated that definite conclusions could not be drawn and that additional research with longer follow-up is required [152].

**Table 3:** Antidepressant medication in Inflammatory Bowel Disease patients.

Study Design	Sample Size	Main Points	Ref.
Retrospective, observational study	29 IBD patients (14 UC, 15 CD)	Antidepressants (80% represented by SSRI) seemed to reduce relapse rates.	[138]
Prospective, observational study	26 CD patients	Fluoxetine (SSRI) was not superior to placebo in maintaining remission.	[153]
Prospective, observational study	67 IBD patients (31 CD, 36 UC)	Antidepressive drug treatment (SSRI 48.4%, SNRI 8.7%, NDRI 12.3%, NaSSA 12.3%, and combination therapies 17.5%) was associated with an improvement in depression, anxiety, QoL, and sexual functioning scores, as well as an improvement in Crohn’s disease activity index.	[139]
Randomized, double-blind, controlled clinical trial	44 IBD patients	Duloxetine (SNRI) was effective to reduce depression, anxiety, and severity of disease symptoms and to increase physical, psychological, and social dimensions of QoL	[144]
A prospective, randomized, double-blind, placebo-controlled clinical trial	45 IBD patients	Venlafaxine (SNRI) significantly improved QoL, anxious and depressive symptoms, and the activity of IBD.	[154]
Population-based cohort study	42,890 IBD patients (69.5% UC; 30.5% CD)	Antidepressant users (SSRI 53.4%, TCA 21.6%, SNRI 14.3%, mirtazapine 8.7%, and other antidepressants 2%) had a significantly lower relapse rate than nonusers, particularly in patients with no use of antidepressants before IBD onset.	[142]
Retrospective cohort study	29,393 IBD patients, (42.2% CD, 57.8% UC)	Antidepressants use SSRI 66.2%, SNRI 13.5%, TCA 10.8%, other antidepressants 9.5%) was independently associated with corticosteroid use, visits, and hospitalizations but was negatively associated with surgery and IBD-related complications.	[146]
Retrospective cohort study	81 IBD patients (58 CD, 23 UC) and 77 IBS patients	TCA led to moderate improvement of residual GI symptoms in IBD patients, particularly in UC pts. This result was similar to IBS patients.	[143]

SSRI, selective serotonin reuptake inhibitor; QoL, quality of life; SNRI, serotonin–noradrenaline reuptake inhibitor; TCA, tricyclic antidepressants; GI, gastrointestinal; CD, Crohn’s disease; UC, ulcerative colitis; IBD, inflammatory bowel disease; IBS, irritable bowel syndrome.

## 5. Conclusions

The intricate connection between IBD and DDs has been increasingly recognized, highlighting the bidirectional relationship and shared biological mechanisms between these conditions. Several key points can be pointed out from the review.

IBD patients exhibit a significantly higher prevalence of psychiatric conditions, such as depression and anxiety, compared with the general population. This association is consistent across various studies and geographic regions, with a substantial percentage of IBD patients experiencing these psychiatric symptoms.

Genetic studies have identified common variants associated with both IBD and depression, indicating a genetic predisposition that may contribute to their co-occurrence. The HPA axis has also emerged as a critical player, as dysregulation within this system could significantly impact stress responses and contribute to the pathogenesis of IBD.

Moreover, immune dysregulation in IBD patients involves various interleukins, such as IL-6, IL-12, IL-17, and TNF-α, which not only exacerbate intestinal inflammation but affect mood and behaviour through their systemic effects. Additionally, gut microbiota dysbiosis, characterized by an imbalance between beneficial and harmful bacteria, is implicated in both IBD and DDs, further supporting the role of the gut–brain axis in these conditions.

The link between IBD and DDs is highlighted by the observation that medications commonly used for treating IBD, including anti-TNFs and anti-integrin alpha4/beta7, have shown efficacy in improving depressive and anxiety symptoms. However, whether these treatments can also induce depressive symptoms as adverse psychiatric events remains to be defined. Moreover, evidence suggests that the use of antidepressants may reduce the risk of developing IBD, highlighting a potential protective effect of these medications.

Further research is needed to clarify the mechanisms through which IBD treatments influence psychiatric symptoms and vice versa. There is a need for longitudinal studies to better understand the temporal relationship between IBD and DDs and to identify potential biomarkers for predicting psychiatric outcomes in IBD patients.

In summary, this review points out the importance of an integrated approach in IBD management, considering both the physical and psychological aspects of the disease. Integrating psychiatric care into the treatment plan for IBD patients could improve overall outcomes and quality of life for this population.

## 6. Future Perspectives

Further research is needed to clarify the mechanisms through which IBD treatments influence psychiatric symptoms and vice versa. Longitudinal studies are essential to better understand the temporal relationship between IBD and DDs, identify potential biomarkers for psychiatric outcomes, and explore the therapeutic potential of microbiome-targeted interventions such as probiotics, prebiotics, or fecal microbiota transplantation. Additionally, incorporating routine psychiatric screening into IBD care pathways may enable earlier identification and management of mood disorders, ultimately improving patient outcomes. From a clinical perspective, it is advisable to establish a tight collaboration between gastroenterologists and mental health professionals to implement integrated care models, facilitate early referral, and consider psychotropic and microbiota-modulating therapies as part of a personalized treatment strategy.

Efforts to develop individualized treatment regimens that account for both intestinal inflammation and mental health status may represent the next frontier in managing complex chronic diseases such as IBD. Future research should also explore the role of novel psychobiotics, dietary interventions, and digital mental health tools tailored to IBD patients.

## 7. Limitations

This review has several limitations that should be acknowledged. First, the heterogeneity among the included studies, in terms of diagnostic criteria, study design, and IBD phenotypes, limits the generalizability of conclusions. Most of the evidence derives from observational and cross-sectional data, which hinders the ability to determine causal relationships. In addition, the potential dual effect of some treatments (e.g., antidepressants reducing inflammation vs. biologics potentially inducing psychiatric side effects) cannot be established in the absence of randomized controlled trials. Finally, while this review focuses on depression and anxiety, other relevant psychiatric conditions, such as cognitive impairment and stress-related disorders, remain underexplored.

## Figures and Tables

**Table 1:** Summary of peripheral and mucosal cytokine levels in the context of inflammatory bowel disease and depressive disorders.

Cytokine	Role	DDs	IBD		References
			CD	UC	
IL-1β	Costimulation in an inflammatory microenvironment	↑	↑	↑	[99,100,101]
IL-2	Promotes T cell proliferation	↑	-	↓	[101,102]
IL-4	Induces B cell activation, IgE switch, and differentiation toward Th2 cells	↓	-	-	[101]
IL-5	Promotes eosinophil growth, differentiation	↑	↑	↑	[101,103]
IL-6	Induces T and B cell growth and differentiation, acute phase production, fever	↑	↑	↑	[99,101,104]
IL-7	Induces growth of preB-cells and preT-cells	↑	-	-	[105]
IL-8	Neutrophils and T cells chemotactic factor.	varies	↑	↑	[101,106,107]
IL-9	Induces mast cell activity, stimulates Th cells	↑	↑	↑	[105,108]
IL-10	Inhibits cytokine synthesis, anti-inflammatory activity	↓	↓	↓	[109,110]
IL-11	Stromal cell regulation of fibrosis	-	↑	varies	[111,112]
IL-12	NK-cell-stimulating factor, activates and proliferates Th1 cells	↑	↑	↑	[101,113]
IL-13	Induces B cell growth and differentiation, induces allergy/asthma	↑	-	-	[101]
IL-15	IL-2-like cytokine, T cell and NK cell growth factor, enhances memory CD8 T cell survival	↑	↑	↑	[105,114]
IL-17A	Proinflammatory, induces cytokine production by epithelia, endothelia, astrocytes, and fibroblasts	↑	↑	↑	[101,115]
IL-18	Induces IFN-γ production by T cells and NK cells, promotes Th1 induction	↑	↑	↑	[101,116]
IL-21	Th17 differentiation, B cell homeostasis	no change	↑	↑	[117,118]
IL-33	Involved in type 2 immunity and allergic airway diseases	↓	↑	↑	[119,120]
TGFβ1	Anti-inflammatory factor, contributing to immune response regulation and overall homeostasis.	no change	↑	↑	[101,121]
TNF	Promotes inflammation, endothelial activation	↑	↑	↑	[99,101,122]
IFN-γ	Induces macrophage activation, increased expression of MHC molecules and antigen processing components, and immunoglobulin class switching, suppresses Th2 cells	varies	↑	↑	[101,123]

↑ increase; ↓ decrease; - no change.

**Table 2:** Impact of medications used for IBD on DDs.

Study Design	Sample Size	Main Points	Ref.
Prospective cohort study	160 IBD patients (49 anti-TNFs, 111 vedolizumab)	VEDO and anti-TNFs were associated with improvement in sleep and mood quality in IBD	[138]
Phase III, randomized, double-blind clinical trial	492 CD patients	At week 56, the group treated with ADA had a statistically significantly lower percentage of patients with depression than the placebo group	[139]
Double-blind, cross-over RCT	104 CD patients	The second most common APE in patients treated with USTE was depression (N = 2; 1.53%)	[136]
Phase III, randomized, parallel-group, double-blind, placebo-controlled trial	967 CD patients	Two patients treated with VEDO developed depression (0.21%), and two VEDO-treated patients (0.21%) expressed suicidality	[137]

IBD, inflammatory bowel disease; TNF, tumour necrosis factor; RCT, randomized controlled trial; CD, Crohn’s disease; APE (adverse psychiatric event); IFX, infliximab; VEDO, vedolizumab; ADA, adalimumab; USTE, ustekinumab.
